# Whole Genome Characterization of the High-Risk Clone ST383 *Klebsiella pneumoniae* with a Simultaneous Carriage of *bla*_CTX-M-14_ on IncL/M Plasmid and *bla*_CTX-M-15_ on Convergent IncHI1B/IncFIB Plasmid from Egypt

**DOI:** 10.3390/microorganisms10061097

**Published:** 2022-05-26

**Authors:** Eva A. Edward, Nelly M. Mohamed, Azza S. Zakaria

**Affiliations:** Department of Microbiology and Immunology, Faculty of Pharmacy, Alexandria University, El-Khartoom Square, Azarita, Alexandria 25435, Egypt; eve.farid@alexu.edu.eg (E.A.E.); nelly.mohamed@alexu.edu.eg (N.M.M.)

**Keywords:** *Klebsiella pneumoniae*, whole genome sequencing, high-risk clone ST383, *bla*_CTX-M-14_, *bla*_CTX-M-15_, convergence of resistance and virulence

## Abstract

Recently, Egypt has witnessed the emergence of multidrug-resistant (MDR) *Klebsiella pneumoniae,* which has posed a serious healthcare challenge. The accelerated dissemination of *bla*_CTX-M_ genes among these MDR *K. pneumoniae*, particularly *bla*_CTX-M-14_ and *bla*_CTX-M-15_, have been noted. In this study, we investigated the occurrence of *bla*_CTX-M-IV_ among *K. pneumoniae* recovered from the laboratory of a major hospital in Alexandria. The 23 tested isolates showed an MDR phenotype and the *bla*_CTX-M-IV_ gene was detected in ≈22% of the isolates. The transformation of plasmids harboring *bla*_CTX-M-IV_ to chemically competent cells of *Escherichia coli* DH5α was successful in three out of five of the tested *bla*_CTX-M-IV_-positive isolates. Whole genome sequencing of K22 indicated that the isolate belonged to the high-risk clone ST383, showing a simultaneous carriage of *bla*_CTX-M-14_ on IncL/M plasmid, i.e., pEGY22_CTX-M-14, and *bla*_CTX-M-15_ on a hybrid IncHI1B/IncFIB plasmid, pEGY22_CTX-M-15. Alignment of both plasmids revealed high similarity with those originating in the UK, Germany, Australia, Russia, China, Saudi Arabia, and Morocco. pEGY22_CTX-M-15 was a mosaic plasmid that demonstrated convergence of MDR and virulence genes. The emergence of such a plasmid with enhanced genetic plasticity constitutes the perfect path for the evolution of *K. pneumoniae* isolates causing invasive untreatable infections especially in a country with a high burden of infectious diseases such as Egypt. Therefore there is an imperative need for countrywide surveillances to monitor the prevalence of these superbugs with limited therapeutic options.

## 1. Introduction

During the current era of antibiotic resistance, *Klebsiella pneumoniae*, a persistent opportunistic nosocomial organism, has been categorized as one of the ESKAPE pathogens which is responsible for almost one-third of the entire Gram-negative-related infections and efficiently “escapes” the effects of antibiotics [1]. Data from the European Antimicrobial Resistance Surveillance Network, from 2005 to 2015, have shown an international increased resistance rate for *K. pneumoniae* towards aminoglycosides, fluoroquinolones, carbapenems, and third-generation cephalosporins. In Mediterranean countries, an endemic prevalence of multidrug-resistant *K. pneumoniae* (MDR-KP) has been recorded [1] and, as a part of this area, Egypt has witnessed a significant spread of MDR-KP with several reports being issued [2,3]. What renders the problem more complicated is that the proper treatment choice for MDR-KP infections is not well determined yet, thus, making the control of such infections a serious challenge for healthcare professionals [4].

Expanded-spectrum cephalosporins are still among the most globally prescribed antimicrobial agents [5]. This extensive usage has been mirrored by a global expansion of extended-spectrum β-lactamase (ESBL)-producing *K. pneumoniae* showing resistance to ceftriaxone, cefotaxime, ceftazidime, and monobactams (as aztreonam), reaching 50% in some countries [4,6]. At a national level, a surveillance study carried out in five hospitals in Cairo, from 1999 to 2000, showed reduced susceptibility of *Klebsiella* spp. isolates to ceftazidime, to approximately 40%, revealing an elevated rate of AmpC and/or ESBL production [7]. In 2016, a nationwide investigation was performed among 91 intensive care units in 28 Egyptian hospitals, and it was reported that the most common pathogen causing healthcare-associated infections was *Klebsiella* spp., of which 42.5% produced ESBLs [8].

In recent years, the repetitive use of cefotaxime and ceftazidime in therapy regimens for infections due to ESBL-producing *K. pneumoniae* has induced the emergence of *K. pneumoniae* harboring the most widespread type of ESBLs, the CTX-M-β-lactamases [6]. Based on their amino acid sequence identities, this group of molecular class A ESBLs has been subdivided into five clusters (from Group I to Group V) all possessing superior hydrolytic activity against ceftriaxone and cefotaxime [9] rather than ceftazidime [6]. Group I comprises CTX-M-1, -3, -10, -11, -12, -15, -22, -23, -28, -29, and -30; Group II includes CTX-M-2, -4, -5, -6, -7, and -20, and Toho-1; Group III consists of CTX-M-8; Group IV comprises CTX-M-9, -13, -14, -16, -17, -18, -19, -21, and -27, and Toho-2; the last group, group V, includes CTX-M-25 and -26 [9]. Massive nosocomial outbreaks due to CTX-M-producing *K. pneumoniae* have been described in different continents including Africa [6,10]. Apparently, highly mobilizable genetic platforms and epidemic spreading of particular strains have fueled this vast and accelerated dispersion of *bla*_CTX-M_ [11]. Additionally, the co-existence of *bla*_CTX-M_ with more resistance determinants has contributed to extensive spreading of CTX-M enzymes, raising the alarm for unmanageable pandemic scenarios [12].

Among the characterized CTX-M enzymes, both CTX-M-14 and CTX-M-15 are the most dominant in the *bla*_CTX-M_ landscape, and are capable of invading both humans and food-producing animals [12]. Since its first recognition in 1997 in China, CTX-M-14 has shown wide dissemination around the globe, not excluding North African countries such as Egypt and Tunisia [11,13]. CTX-M-15 was originally identified among enteric isolates at an Indian hospital in 1999 [12], but very rapidly reached a worldwide prominence covering areas in the Middle East region and Arab countries such as Egypt, the United Arab Emirates, and Kuwait [14,15]. The dominance of *bla*_CTX-M-14_ and *bla*_CTX-M-15_ among other *bla*_CTX-M_ is endorsed by their association with insertion sequences (IS) and their primary location on conjugative plasmids of prevalent F-type trafficking freely among bacterial hosts [16]. Given the fact that the most common hypervirulence-associated genes in *K. pneumoniae* isolates, such as *iuc* (aerobactin siderophore) and *rmpA2* (hypermucoidy) loci, are mobilized on virulence plasmids, evidence of the sporadic convergence of plasmids carrying *bla*_CTX-M_ and virulence determinants within a single genetic environment is emerging nowadays globally [17] and nationally [18]. The ultra-mosaic nature of *K. pneumoniae* plasmids paves the way for this convergence creating antimicrobial resistant (AMR)-hypervirulent vectors that could spread easily, expanding the picture to a new magnitude of hard-to-treat infections and fatal outbreaks [17]. In this study, we present the molecular characterization of a *K. pneumoniae* Egyptian isolate of the recently emerging high-risk clone ST383 carrying *bla*_CTX-M-14_ and *bla*_OXA-48_ on an IncL/M plasmid and simultaneously harboring *bla*_CTX-M-15_ and *bla*_NDM-5_ on a mosaic IncFIB/IncHI1B plasmid converging resistance and virulence genes.

## 2. Materials and Methods

### 2.1. Bacterial Strains Collection and Identification

Twenty-three non-duplicate clinical isolates of *Klebsiella* spp. showing resistance to third-generation cephalosporins were collected from the laboratories of Mabaret-Al Asafra Hospitals from July to August 2020. Mabaret Al-Asafra Hospitals serve a large proportion of patients through 7 satellite branches of laboratories in Alexandria, Egypt. These isolates were numbered from K1 to K23. The clinical origins of these isolates were as follows: blood (*n* = 13), swab (*n* = 3), aspirate (*n* = 2), mini-bronchoalveolar lavage (*n* = 1), bronchoalveolar lavage (*n* = 1), pleural fluid (*n* = 1), urine (*n* = 1), and endotracheal tube (*n* = 1). All isolates were preserved as frozen stocks in Luria-Bertani broth (LB, HiMedia, Mumbai, India) containing 15% glycerol at −20 °C. A fresh culture was obtained using a subculture of the isolates on MacConkey agar (HiMedia, Mumbai, India) for 24 h at 37 °C before use. The collected isolates were identified at the species level using a VITEK^®^ 2 Compact System in conjunction with a 2 GN Identification Card (bioMérieux, Marcy-L’Etoile, France), according to the manufacturer’s instructions.

### 2.2. Antimicrobial Susceptibility Testing and Calculation of Resistance Score

The susceptibilities of the 23 *Klebsiella* spp. isolates to ticarcillin, ticarcillin/clavulanate, piperacillin, piperacillin/tazobactam, ceftazidime, cefepime, aztreonam, imipenem, meropenem, amikacin, gentamicin, tobramycin, ciprofloxacin, and sulfamethoxazole/trimethoprim were determined using a VITEK^®^ 2 Compact system with an updated Advanced Expert System (AES) (bioMérieux, Marcy-L’Etoile, France) and an N222 sensitivity card, according to the manufacturer’s instructions. A resistance score, defined as the number of antimicrobial agents to which a tested isolate showed resistance, was determined for each isolate. Any resistant call was given a score of 1, while an intermediate resistance to the tested agent was given a score of 0.5 [5].

### 2.3. Determination of the Minimum Inhibitory Concentration (MIC) of Cefotaxime against Klebsiella *spp.* Isolates

The MIC of cefotaxime (Claforan^®^, as cefotaxime sodium, Sanofi, Egypt), a third-generation cephalosporin, was determined against collected *Klebsiella* spp. isolates following the broth microdilution method in triplicate. The results were interpreted according to the guidelines of the Clinical and Laboratory Standards Institute (CLSI, Malvern, PA, USA, 2021) which considers isolates with a cefotaxime MIC value of ≥4 µg/mL to be resistant [19]. *Escherichia coli* ATCC 25922 was included as a quality control strain.

### 2.4. Polymerase Chain Reaction (PCR) Detection of bla_CTX-M-IV_

Colony PCR was performed to detect the genes coding for the CTX-M enzymes of Group IV among the collected isolates [20]. The following primers, obtained from Willowfort, United Kingdom, were used: *bla*_CTX-M-IV_-forward (5′-GACAAAGAGAGTGCAACGGATG-3′) and *bla*_CTX-M-IV_-reverse (5′-TCAGTGCGATCCAGACGAAA-3′) [21]. The obtained PCR products were separated by gel electrophoresis in the presence of 100 bp DNA ladder (GeneDireX^®^, Miaoli, Taiwan) used as a marker. The bands were visualized on a 254 nm UV transilluminator (Entela UVP, CA, USA).

### 2.5. Isolation of Plasmids Harboring bla_CTX-M-IV_ and Plasmid Transformation

Plasmid DNA was extracted from *bla*_CTX-M-IV_-positive isolates using a QIAGEN Plasmid Mega Kit^®^ (QIAGEN, Venlo, The Netherlands), according to the manufacturer’s instructions. Transformation of plasmids harboring *bla*_CTX-M-IV_ to *E. coli* DH5α chemically competent cells was performed using the heat shock technique according to Tu et al. [22], with some modifications. Transformants were selected on MacConkey agar plates supplemented with cefotaxime (4 μg/mL), tested for susceptibility to cefotaxime, ceftazidime, cefepime, imipenem, gentamicin, ciprofloxacin, doxycycline, amoxicillin/clavulanate, and sulfamethoxazole/trimethoprim using the disk diffusion method, and the results were interpreted according to CLSI 2021 [19]. The MIC of cefotaxime was determined for recipient and representative transformants following the broth microdilution method, as described earlier. The presence of *bla*_CTX-M-IV_ in transformants was confirmed by PCR amplification using the previously mentioned primers.

### 2.6. Whole Genome Sequencing (WGS) of K. pneumoniae K22 Isolate

After extraction of the genomic DNA from the *K. pneumoniae* strain K22, its integrity was checked by running an agarose gel electrophoresis and it was quantified using a Quant-iT™ PicoGreen^®^ dsDNA assay kit (Invitrogen, Cat. No. P11496). The sequencing libraries were prepared according to the manufacturer’s instructions for TruSeq^®^ Nano DNA Library Prep kits (Illumina, Inc., San Diego, CA, USA) and following the TruSeq Nano DNA Sample Preparation Guide (Part # 15,041,110 Rev. D). Briefly, fragmentation of 100 ng of genomic DNA was performed using Adaptive Focused Acoustics^®^ technology (AFA^®^; Covaris, MA, USA) and the fragmented DNA was end-repaired to create 5′-phosphorylated, blunt-ended dsDNA molecules which were size selected using the bead-based method. The purified libraries were quantified using qPCR, according to the qPCR Quantification Protocol Guide (KAPA Library Quantification Kits for Illumina sequencing platforms) and qualified using an Agilent 2200 TapeStation system (Agilent Technologies, Santa Clara, CA, USA). Then, paired-end (2 × 150 bp) sequencing was performed by Macrogen (Seoul, Korea).

### 2.7. Bioinformatics Analysis

The obtained raw reads were trimmed and de novo assembled using SPAdes software (v3.15.3) (https://cab.spbu.ru/software/spades/) (accessed on 11 December 2021) with default settings. A low k-mer (k = 31) and a high k-mer (k = 127) were applied to build assembly graphs where low k-mers allowed the discovery of variants at relatively lower coverage, while genome complexity and large structural variations were more approachable at high k-mers. Then, assemblies were filtered, maintaining nodes of more than 500 bp. The obtained scaffolds were analyzed utilizing the pipelines of the Center for Genomic Epidemiology (CGE) (http://www.genomicepidemiology.org/) (accessed on 13 December 2021) to identify antimicrobial resistance genes (ResFinder v4.1), to perform in silico multi-locus sequencing typing (MLST v2.0) and plasmid typing (PlasmidFinder v2.1). Detection of virulence genes, heavy metal resistance genes, and typing of *wzc*- and *wzi*-alleles were achieved through the Institut Pasteur website (https://bigsdb.pasteur.fr/klebsiella/) (accessed on 25 January 2022). Capsule type and O-antigen locus type were determined using the K-PAM in silico diagnostic tool (https://www.iith.ac.in/K-PAM/pim.html) (accessed on 2 February 2022).

To determine the *bla*_CTX-M-14_- and *bla*_CTX-M-15_-harboring plasmid sequence, assembled contigs from strain K22 were mapped against *K. pneumoniae* (taxid:573) using BLASTn (https://blast.ncbi.nlm.nih.gov/Blast.cgi) (accessed on 1 February 2022). The complete sequences of the generated plasmids pEGY22_CTX-M-14 and pEGY22_CTX-M-15 in strain K22 were assembled by scaffolding several nodes while any overlap regions were manually inspected. SnapGene v6.0.2 (Insightful Science, San Diego, CA, USA, www.snapgene.com) (accessed on 24 February 2022) was used for the drawing and the annotation of pEGY22_CTX-M-14 and pEGY22_CTX-M-15 plasmids. Exploiting CGE pipelines, PlasmidFinder v2.1 (https://cge.cbs.dtu.dk//services/PlasmidFinder/) (accessed on 8 February 2022) was used to detect the plasmid incompatibility (Inc) groups, while IS elements of the plasmids were identified using MobileElementFinder v1.0 (https://cge.cbs.dtu.dk/services/MobileElementFinder/) (accessed on 8 February 2022).

### 2.8. Analysis of pEGY22_CTX-M-14 and pEGY22_CTX-M-15 to Closely Related Plasmids

Sequences of related plasmids with similar Inc groups were mined from the NCBI (https://www.ncbi.nlm.nih.gov/) (accessed on 1 February 2022). Comparisons between each of pEGY22_CTX-M-14 and pEGY22_CTX-M-15 plasmids with the related plasmids were performed using the BLAST Ring Image Generator (BRIG) tool (https://sourceforge.net/projects/brig/) (accessed on 22 February 2022), and BLASTn (https://blast.ncbi.nlm.nih.gov/Blast.cgi) (accessed on 22 February 2022).

## 3. Results and Discussion

### 3.1. Antimicrobial Resistance Profile and Resistance Score

The identity of 23 clinical isolates of *Klebsiella* spp. was confirmed as *K. pneumoniae* subsp. *pneumoniae* using the VITEK^®^ 2 GN ID card. Their antimicrobial susceptibility pattern against 15 antibiotics revealed that all isolates exhibited an MDR phenotype, since they were resistant to at least three antibiotic classes including penicillins, cephems, and aminoglycosides, according to the criteria proposed by Magiorakos et al. [23]. Nine isolates (39.1%) exhibited resistance to all tested antibiotics, scoring 15 in the calculated resistance score (Table 1). The availability of antibiotics without prescriptions in a lower middle-income country such as Egypt and the lack of strict antibiotic policies are the major driving forces accelerating the spread of these MDR strains [5]. All tested isolates were resistant to piperacillin, ticarcillin, cefotaxime, and ceftazidime (Supplementary Table S1). Although previous studies from Egypt have documented the ability of imipenem to retain its resilience [2,3], 18 isolates (78.3%) were found to be carbapenem resistant. High resistance rates, exceeding 70%, were detected to β-lactam/β-lactamase combinations, monobactam, fourth-generation cephalosporin, aminoglycosides, and antifolates. Similar elevated levels of resistance to frontline antibiotics among *K. pneumoniae* isolates were recently reported from institutional facilities in both Cairo and Alexandria [24,25]. Less resistance was evident to ciprofloxacin as compared with other tested antibiotics, an observation supported by other researchers from the Upper Egypt sector [26]. Among fluoroquinolones, levofloxacin is preferred over ciprofloxacin when empiric therapy is necessary, as recommended by Infectious Diseases Society of America (IDSA) guidelines implemented in Egyptian healthcare establishments. This decline in the prescription levels of ciprofloxacin might be the reason behind the lower resistance rates detected for this antibiotic.

### 3.2. Molecular Identification of bla_CTX-M-IV_

In 2021, Palmieri et al. declared that the most prevalent CTX-M-type ESBL, in a large longitudinal collection of *K. pneumoniae* strains isolated over 15 years in China, was a member of group IV, i.e., *bla*_CTX-M-14_ [27]. There is a paucity of information in Egypt on the characterization of different CTX-M enzymes in *K. pneumoniae* isolates, yet an earlier study in 2009 reported the occurrence of *bla*_CTX-M-14_ in *K. pneumoniae* isolated from patients in an ICU ward [13]. Moreover, CTX-M-14 enzymes are highly malleable with a broad ability for evolution. Many of the novel alleles of *bla*_CTX-M_ are derived from CTX-M-14-β-lactamase genes via homologous recombination [11]. Inspired by both studies and understanding the importance of CTX-M-14, we decided to focus on the molecular detection of *bla*_CTX-M_ belonging to group IV in our collection of *K. pneumoniae* isolates. A PCR analysis identified an amplicon of 501 bp corresponding to *bla*_CTX-M-IV_ in 5/23 (21.7%) of the isolates (Supplementary Figure S1). These isolates were: K1, K7, K14, K22, and K23. In 2009, a study in Korea described a higher prevalence rate of *bla*_CTX-M-IV_ reaching 46% among 37 *K. pneumoniae* strains isolated from a children’s hospital in Seoul [28]. Most of the studies originating from Egypt that contain data about the prevalence of *bla*_CTX-M_ in *K. pneumoniae* isolates have used universal *bla*_CTX-M_ primers which do not differentiate among different alleles of *bla*_CTX-M_ [24,26]. Nevertheless, Abdelwahab et al., in 2021, characterized the whole genome of four *K. pneumoniae* isolates and reported that three-quarters of the isolates harbored *bla*_CTX-M-14_, a member of *bla*_CTX-M-IV_ [18]. Another study, in 2021, which included nine Egyptian hospitals, documented that 30.8% of MDR *K. pneumoniae* expressed *bla*_CTX-M-9_, another member of *bla*_CTX-M-IV_ [29]. In the Middle East, particularly in Saudi Arabia, the occurrence rate of *bla*_CTX-M-14_ among *K. pneumoniae* isolates has fluctuated. In 2011, *bla*_CTX-M-14_ was detected in 1.8% of 430 *K. pneumoniae* isolates [30]. However, in 2018, the prevalence rate reached 21% in a tertiary hospital in Riyadh, a percentage determined in our study [31]. The *bla*_CTX-M-IV_-positive isolates were selected for further investigations.

### 3.3. Purification of bla_CTX-M-IV_-Encoding Plasmids and Transformation Experiment

To investigate whether *bla*_CTX-M-IV_ genes were located on plasmids and whether the transfer of these genes contributed to the reduced susceptibility of the recipient *E. coli* DH5α towards antibiotics, plasmids’ DNAs were extracted from *bla*_CTX-M-IV_-positive isolates by alkaline lysis and transformed to chemically competent cells of *E. coli* DH5α. Transformation was successful in three out of five of the tested isolates and the movements of *bla*_CTX-M-IV_ genes were verified in the generated transformants by determining the MIC of cefotaxime, the disk diffusion method, and the PCR amplification of *bla*_CTX-M-IV_ using specific primers described earlier (Table 2). The resistance phenotype of the transformants included resistance to amoxicillin/clavulanate, cefotaxime, and ceftazidime. The values of cefotaxime MIC for the transformants increased from 32- to 128-fold as compared with the recipient, inferring that *bla*_CTX-M-IV_ genes were positioned on self-transferable plasmids in K7, K14, and K23 isolates. Interestingly, *bla*_CTX-M-IV_ genes were not mobilized by transformation in isolates K1 and K22 despite several attempts. Therefore, it was suggested that the *bla*_CTX-M-IV_-bearing plasmids in these strains were non-self-transmissible. A similar situation was encountered in a previous study, from Egypt, by Kalaf et al. [13] who reported that *bla*_CTX-M-14_, a member of *bla*_CTX-M-IV_, was not transferable. However, the authors did not elaborate the reasons behind unsuccessful transmission. Consequently, we aimed, in our study, to shed light on the genomic features of the *K. pneumoniae* isolate which failed to transform its plasmids to the chemically competent cells of *E. coli* DH5α and to explore the genetic characteristics of these plasmids using WGS analysis.

### 3.4. Whole Genome Sequencing of K. pneumoniae Isolate K22

Two isolates investigated in the present study, K1 and K22, showed unsuccessful transformation results; both isolates originated from blood source and scored 15 in the calculated resistance score (Table 1). As a representative of both isolates, isolate K22 was selected for WGS analysis using the Illumina platform. The de novo assembled complete genome of K22 was distributed in 295 scaffolds, had an N50 of 88735, and comprised a chromosome of 4,955,068 bp with an overall G+C content of 57.7%. The statistics of sequence assembly generated through WGS are described in Supplementary Table S2. Genotyping of K22 indicated that the isolate belonged to the sequence type (ST) ST383 according to the MLST allelic profile which uses the sequences of the seven housekeeping genes *gapA*, *infB*, *mdh*, *pgi*, *phoE*, *rpoB*, and *tonB*, with assigned allele numbers 2, 6, 1, 3, 8, 1, and 18, respectively. Based on the *wzi* gene DNA sequences and *wzc* typing, K22 strain possessed wzi705-wzc50 allele, and its serotype was determined as K51 and O1 referring to the capsular polysaccharide (K antigen) and the lipopolysaccharide (O antigen), two important virulence factors essential for the differentiation of *K. pneumoniae* isolates (Table 3). ST383 is an emerging high-risk clone that has established global dissemination with reports being issued from different geographical regions including Greece, UK, China, and Egypt [27,29,32,33,34]. This high-risk clone is imposing an alarming situation reflected by its carriage of carbapenemase-encoding genes of different types such as *bla*_OXA-48_ and *bla*_NDM_, thus, limiting the available frontline treatment options [27]. The results of the K22 resistome were consistent with this remark, revealing the isolate’s possession of carbapenem resistance mediated by both *bla*_NDM-5_ and *bla*_OXA-48_ genes. Furthermore, the genome of K22 clearly displayed an MDR genotype, carrying genes responsible for resistance to aminoglycosides (*aph(6)-Id, aph(3″)-Ib, aph(3′)-VIb, aadA1, aph(3′)-VI, aac(6′)-Ib,* and *armA*), amphenicols (*catA1* and *catB3*), sulphonamides (*sul1* and *sul2*), fluoroquinolones (*aac(6′)-Ib-cr*, *qnrS1*, *oqxA*, and *oqxB*), tetracycline (*tetA*), macrolides and lincosamides (*mphE*, *msrE*, and *mphA*), fosfomycin (*fosA*), and trimethoprim (*dfrA5*). ResFinder identified several β-lactamase genes including *bla*_SHV-26_, *bla*_TEM-1_, *bla*_OXA-1_, and *bla*_OXA-9_. Two CTX-M-β-lactamases were detected in isolate K22, *bla*_CTX-M-14b_ (Group IV) and *bla*_CTX-M-15_ (Group I) (Table 3). This finding explains the phenotypic resistance of K22, and the high resistance score assigned for this isolate.

Although the combination of resistance and virulence genes is known to be largely restricted to hypervirulent clones, isolate K22, belonging to the “non-hypervirulent” type [35], was heavily shaped by a variety of virulence factors-encoding genes. Among the siderophores, K22 isolate expressed the yersiniabactin cluster (*irp1*, *fyuA*, and *ybtAEPQSTUX*) and the aerobactin synthetase gene cluster (*iutA* and *iucABCD*). These high-affinity iron acquisition systems tend to counteract the restriction of iron bioavailability, a traditional host defense mechanism against bacterial invasion [36]. The capsular polysaccharide synthesis regulator *rmpA* and its homologue *rmpA2* provide the hypermucoviscous phenotype in the producing strain and coordinate the production of its capsule, a main virulence factor enabling the isolate’s evasion from phagocytosis, complement, antimicrobial peptides, and specific antibodies [37]. An additional regulator of capsule expression, *rmpC*, the mutation of which has been proven to decrease capsule production in mutant strains, was located as well in K22 isolate [38]. Genes coding for type 3 fimbrial adhesins (*mrkABDFHIJ*), mediating enhanced biofilm formation on abiotic surfaces [37], were detected in K22 isolate. Heavy metal resistance loci were identified on the genome of the K22 isolate coding for tellurite (*terABCDEWXYZ*), silver (*silABCEFGPRS*), copper (*pcoABCDRSE*), and arsenic (*arsABCDR*) resistance (Table 3). PlasmidFinder identified eight plasmid replicon types: Col(KPHS6), ColRNAI, Col440II, IncFIB_K_, IncFII_K_, IncFIB, IncHI1B, and IncL/M (Table 3).

### 3.5. Characterization of pEGY22_CTX-M-14 and Its Similarity to IncL/M Published Plasmids

**Figure 1:**
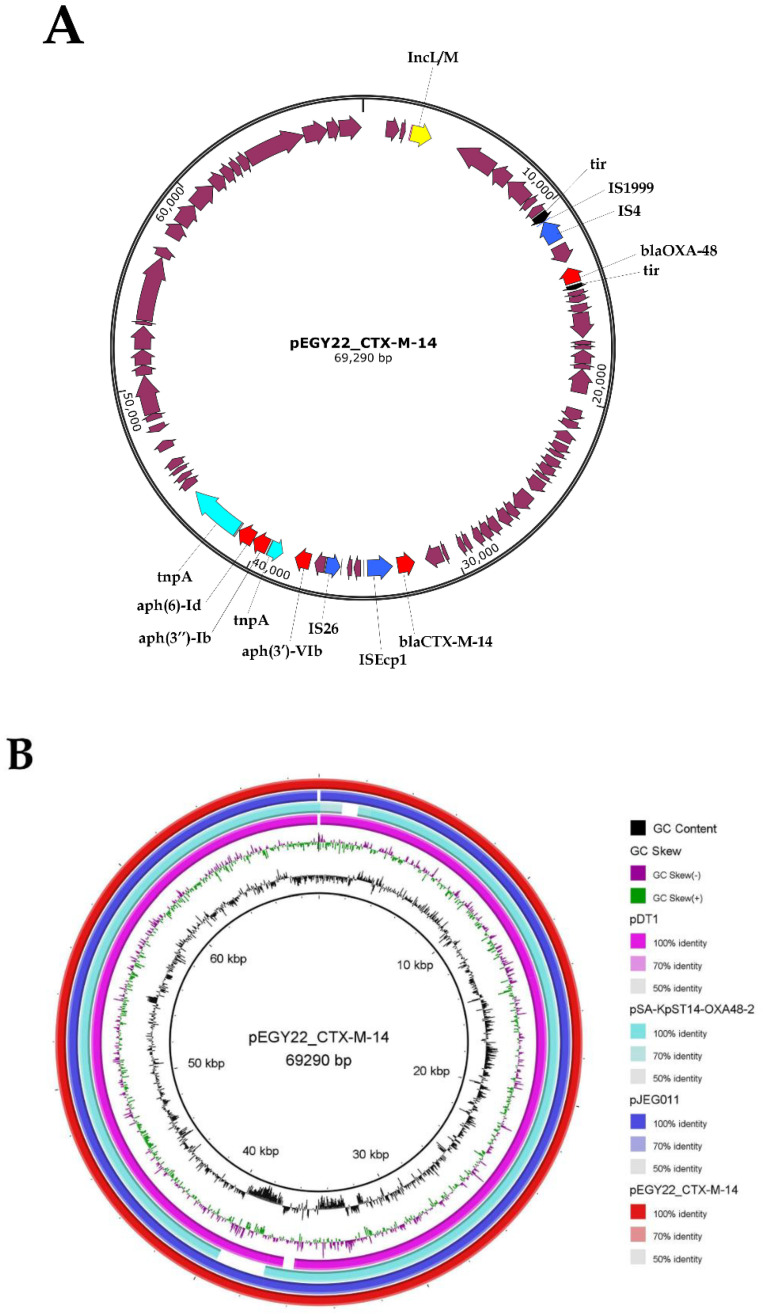
(**A**) Schematic genetic representation of pEGY22_CTX-M-14 plasmid harbored by the *K. pneumoniae* K22 strain. The arrows indicate open reading frames (ORFs) with magenta, aqua, blue, yellow, black, and red representing functional ORFs, transposons, insertion sequence elements, plasmid replicon type, *tir* gene, and antimicrobial resistance genes, respectively; (**B**) BRIG visualization comparing IncL/M-*bla*_CTX-M-14_-positive *K. pneumoniae* plasmids. Circles from inside to outside show the coding sequencing region of pDT1 (NZ_CP019078.1), pSA-KpST14-OXA48-2 (NZ_CP071281.1), pJEG011 (KC354801.1), and pEGY22_CTX-M-14 (ON261190). GC skew (dark green and magenta) and GC content (black) are represented in the inner circles. Genomic regions covered by BLASTn are represented by a solid color in concentric rings (with varying color degrees depending on percentage identity), whereas white gaps indicate genomic regions not covered by BLASTn.

The detected *bla*_CTX-M-14b_ gene was found to reside on an IncL/M plasmid which was 69,290 bp in length, contained 65 CDS, and had an average G+C content of 51%. The plasmid denoted as pEGY22_CTX-M-14 (GenBank accession ON261190) encoded, in addition to *bla*_CTX-M-14b_, aminoglycoside resistance genes (*aph(6)-Id, aph(3″)-Ib,* and *aph(3′)-VIb*), and the *bla*_OXA-48_ carbapenemase gene. IncL/M plasmids represent an emerging threat since they are currently identified to be a source of class D carbapenemase, *bla*_OXA-48_, and responsible for the worldwide dissemination of *bla*_CTX-M_ [39]. Isolate bearing such plasmid becomes resistant to all types of β-lactams and carbapenems [39], a situation that we encountered in the current study. In silico analysis of the genetic context of *bla*_CTX-M-14b_ revealed an upstream location of IS*Ecp1*, one of the most detected IS elements in the genetic environment of the *bla*_CTX-M-14_ gene. IS*Ecp1* implements the mobilization of *bla*_CTX-M-14_ among plasmids, transposons, and integrons, and acts as a promotor upregulating the expression of this gene [40] (Figure 1A). The genetic environment of *bla*_CTX-M-14b_ included additional mobile genetic elements, IS*26*, Tn2*tnpA*, and a truncated Tn2*tnpA*, all located within a 12 kb segment encoding *bla*_CTX-M-14b_ and aminoglycoside resistance genes, suggesting that this resistance segment has been acquired by transposition mechanism. The GenBank databases reveal that plasmids of IncL/M backbone had evolved through the sequential acquisition of resistance genes and IS, considering that the prototype of this Inc group, isolated from *Erwinia amylovora*, neither possessed genes of resistance nor IS [41]. IS*1999* and IS*4* were found in the proximity of *bla*_OXA-48_ carbapenemase (Figure 1A). This may lead to speculate that a sequence of independent acquisitions had been initiated by the integration of IS*1999*-IS*4*-*bla*_OXA-48_ into the *tir* gene which encoded a transfer inhibitory protein [41], while a separate recombination event resulted in the insertion of the large resistance segment encoding *bla*_CTX-M-14b_ and aminoglycosides resistance genes. This could explain the distant location of *bla*_OXA-48_ from this resistance region (Figure 1A). Furthermore, recent findings have shown that bacteriophages play a role in the dissemination of AMR genes among bacterial species where phages are considered to be reservoirs for these genes transferring them to bacterial hosts, and in turn, promoting their own dissemination and survival [42]. Zhou et al. detected, in their study, an *mcr-1*-carrying P7 phage-like plasmid isolated from a clinical *K. pneumoniae* strain and described these phages as new vehicles responsible for the spread of resistance in China [43]. The phage-mediated transduction, as a major driver of horizontal transfer of AMR genes especially those conferring resistance to aminoglycosides, β-lactams (including *bla*_CTX-M-9_, another member of group IV *bla*_CTX-M_), chloramphenicol, or tetracycline had been reported by other authors [42]. Despite the self-conjugative nature of IncL/M plasmids, these plasmid replicons were previously documented to have a low in vitro conjugation frequency rate and their loss during transformation has been reported by other authors [44]. This might be the reason why we were unable to detect the transformants for K22 strain.

The closest matching plasmids to pEGY22_CTX-M-14 from the publicly available database were pJEG011 (GenBank accession KC354801.1) recovered from the *K. pneumoniae* ST101 strain isolated from an ICU patient in Australia [45], and pDT1 (GenBank accession NZ_CP019078.1) recovered from the *K. pneumoniae* ST383 strain isolated from an ICU patient residing in Germany [46]. Both mentioned plasmids exhibited a very high similarity to our plasmid (99.9% nucleotide identity and 99% sequence length) as detected by BLASTn tool and visualized by BRIG. In addition, pEGY22_CTX-M-14 revealed a high similarity (99.9% nucleotide identity and 91% sequence length) to pSA-KpST14-OXA48-2 (GenBank accession NZ_CP071281.1) recovered from *K. pneumoniae* ST14 isolated from the Middle East region, specifically, from Saudi Arabia [47], implicating a geographical spread of this plasmid replicon (Figure 1B).

### 3.6. Convergence of Virulence and Resistance in Plasmid pEGY22_CTX-M-15

**Figure 2:**
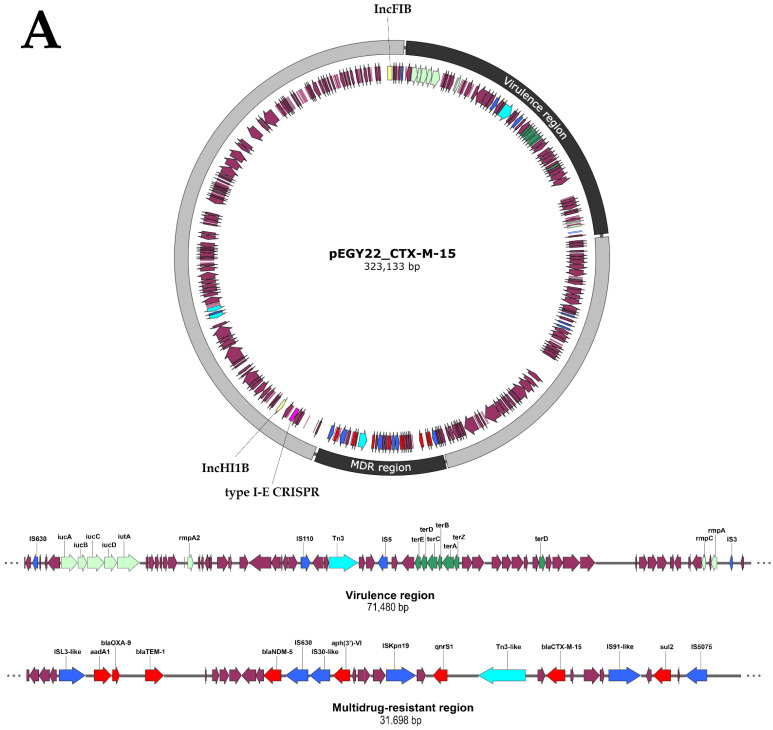

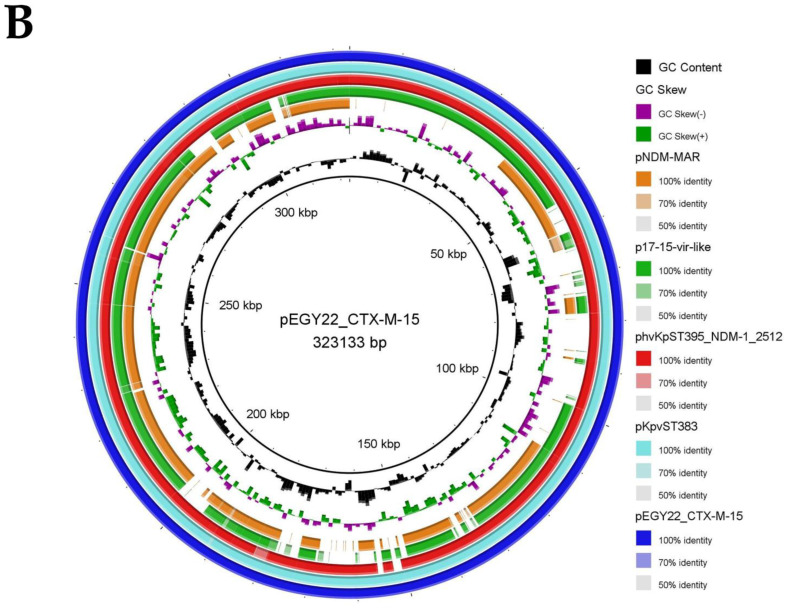
(**A**) Schematic genetic diagram of pEGY22_CTX-M-15 convergent plasmid harbored by the *K. pneumoniae* K22 strain showing a detailed representation of multidrug-resistant and virulence regions. The arrows indicate ORFs with magenta, light green, dark green, aqua, blue, yellow, pink, and red representing functional ORFs, virulence factor genes, heavy metal resistance genes, transposons, insertion sequence elements, plasmid replicon type, CRISPR array, and antimicrobial genes, respectively; (**B**) BRIG visualization comparing IncHI1B/IncFIB-*bla*_CTX-M-15_-positive *K. pneumoniae* plasmids. Circles from inside to outside show the coding sequencing region of pNDM-MAR (JN420336.1), p17-15-vir-like (MN956836.1), phvKpST395_NDM-1_2512 (MW911670.1), pKpvST383 (CP034201.2), and pEGY22_CTX-M-15 (ON261191). GC skew (dark green and magenta) and GC content (black) are represented in the inner circles.

The *bla*_CTX-M-15_ gene was borne by a megaplasmid of 323,133 bp in length, encoding 347 CDS with an average G+C content of 51%. The plasmid designated pEGY22_CTX-M-15 (GenBank accession ON261191) was found to be a mosaic plasmid formed by the fusion of two different plasmid backbones: IncHI1B/IncFIB backbones. Mosaic plasmids created upon fusion of diverse backbones such as IncHI1B/IncFIB, IncFIB_K_/IncHI1B, IncFIB/IncR have been previously documented, mostly in Asia, specifically in China and India [48,49]. Sequence analysis and annotation revealed that plasmid pEGY22_CTX-M-15, apart from encoding *bla*_CTX-M-15_, possessed an MDR region of about 32 kb, harboring seven resistance genes: *bla*_NDM-5_, *bla*_OXA-9_, *bla*_TEM-1_, *aadA1, aph(3′)-VI, qnrS1*, and *sul2* (Figure 2A). The co-existence of *bla*_NDM-5_ and *bla*_CTX-M-15_ compromises significantly the available therapeutic options, as NDM-5 is not inhibited by avibactam, rendering the use of ceftazidime/avibactam profitless, while aztreonam, an agent stable to hydrolysis by NDM, is hydrolyzed by CTX-M-15 [50]. The integration of this MDR cassette into pEGY22_CTX-M-15 must have been facilitated by the presence of two IS, IS*5075* and IS*L3-like*, flanking this cassette (Figure 2A). In addition, the MDR region of pEGY22_CTX-M-15 contained five mobile genetic elements (IS*630*, IS*30-like*, IS*Kpn19*, IS*91-like*, and Tn3-like) distributed throughout the region. Previous studies have demonstrated that resistance loci containing IS elements can act as hotspots attracting further resistance genes and consequently generating novel MDR regions [34]. IS*91-*like and Tn3-like elements were located upstream and downstream of *bla*_CTX-M-15_, respectively, suggesting their role in the mobilization of this gene. A total of sixteen mobile genetic elements were depicted on pEGY22_CTX-M-15 (Figure 2A). These elements presumably play a chief role in the creation of the mosaic nature of megaplasmids through frequent genetic transposition and eventually result in a better adaptation of the plasmid to the bacterial host [17]. Plasmid pEGY22_CTX-M-15 clearly demonstrated the phenomenon of convergence where the MDR region was overlapped with a region encoding a set of virulence genes creating a plasmid of enhanced genetic plasticity. The convergent plasmid harbored the ferric aerobactin receptor and aerobactin siderophore (*iutA* and *iucABCD*), the regulators of mucoid phenotype (*rmpA*, *rmpA2,* and *rmpC*), and the tellurite resistance operon *terABCDEWXYZ*. The occurrence of heavy metal resistance in an isolate of clinical origin is likely to provide the isolate with additional survival parameters in ecological niches apart from hospital settings [48]. In Egypt, a country characterized by its high AMR rates, the convergence of virulence and MDR among *K. pneumoniae* isolates poses a potential healthcare hazard since selection for MDR plasmid-carrying isolates by relevant antibiotics will simultaneously select for virulence characteristics rendering the containment of such isolates very challenging.

### 3.7. Similarity of Plasmid pEGY22_CTX-M-15 to Other Published Plasmids

The sequence of pEGY22_CTX-M-15 plasmid detected in the present study was compared to the closest matching IncHI1B/IncFIB *bla*_CTX-M-15_-carrying plasmids from the global database (Figure 2B). The genomic alignment revealed that pEGY22_CTX-M-15 shared a striking sequence similarity (99.9% nucleotide identity and 99% sequence length) to the IncHI1B/IncFIB *bla*_CTX-M-15_/*bla*_NDM-5_-bearing plasmid, pKpvST383 (GenBank accession CP034201.2), recovered from the *K. pneumoniae* ST383 strain isolated from a patient in London in 2019. The patient suffered from bacteremia, sepsis, multi-organ failure, and subsequently died [51]. It showed a very high similarity (99.9% nucleotide identity and 98% sequence length) as well to a large hybrid plasmid, phvKpST395_NDM-1_2512 (GenBank accession MW911670.1), carrying resistance to carbapenem/CTX-M-15 and hypervirulent genes identified in *K. pneumoniae* ST395 recovered from a patient admitted to ophthalmology unit in a hospital in Saint Petersburg, Russia [52]. The pEGY22_CTX-M-15 plasmid displayed a 98% similar identity to p17-15-vir-like (GenBank accession MN956836.1) and pNDM-MAR plasmid (GenBank accession JN420336.1) with a query cover of 75% and 62%, respectively. The plasmids were both recovered from *K. pneumoniae* ST15 strains, the former being isolated from China while the latter was isolated from Morocco [53,54]. Plasmid pNDM-MAR is the first reported IncHI1B/IncFIB multireplicon plasmid harboring both *bla*_CTX-M-15_ and *bla*_NDM-1_. Considering that this plasmid was identified in Morocco, it might be speculated that a geographical spread of this mosaic plasmid occurred in the Mediterranean basin reaching Egypt [54].

## 4. Conclusions

In conclusion, here, we provide a detailed characterization of *K. pneumoniae* isolate belonging to the high-risk clone ST383 isolated from Egypt, which coharbors the two most dominant *bla*_CTX-M_: *bla*_CTX-M-14_ carried on an IncL/M plasmid and *bla*_CTX-M-15_ located on a hybrid IncHI1B/IncFIB plasmid, displaying convergence of MDR and virulence genes. The emergence of these mosaic structure plasmids with enhanced genetic plasticity enabling simultaneous resistance and virulence within a single vector constitutes the perfect path for the evolution of *K. pneumoniae* isolates causing invasive untreatable infections. The situation in Egypt seems to reflect a global scenario necessitating an imperative countrywide surveillance to closely monitor the prevalence of these superbugs with limited therapeutic options.

## Figures and Tables

**Table 1:** Resistance score of the tested *K. pneumoniae* isolates, their clinical source, and their resistance profile.

*K. pneumoniae* Isolates	Source ^a^	Resistance Score ^b^	Resistance Profile ^c^
**K1**	Blood	15	CTX, TIC, TIM, PIP, TZP, CAZ, FEP, ATM, IPM, MEM, AMK, GEN, TOB, CIP, SXT
**K2**	Pleural fluid	15	CTX, TIC, TIM, PIP, TZP, CAZ, FEP, ATM, IPM, MEM, AMK, GEN, TOB, CIP, SXT
**K3**	Blood	13	CTX, TIC, TIM, PIP, TZP, CAZ, FEP, IPM, MEM, AMK, GEN, TOB, SXT
**K4**	BAL	15	CTX, TIC, TIM, PIP, TZP, CAZ, FEP, ATM, IPM, MEM, AMK, GEN, TOB, CIP, SXT
**K5**	ETT	13	CTX, TIC, TIM, PIP, TZP, CAZ, FEP, ATM, IPM, MEM, GEN, TOB, SXT
**K6**	Blood	10	CTX, TIC, PIP, CAZ, FEP, ATM, **AMK**, GEN, TOB, **CIP**, SXT
**K7**	Blood	14	CTX, TIC, TIM, PIP, TZP, CAZ, FEP, ATM, IPM, MEM, AMK, GEN, TOB, CIP
**K8**	mini-BAL	15	CTX, TIC, TIM, PIP, TZP, CAZ, FEP, ATM, IPM, MEM, AMK, GEN, TOB, CIP, SXT
**K9**	Blood	14	CTX, TIC, TIM, PIP, TZP, CAZ, FEP, ATM, IPM, MEM, AMK, GEN, TOB, CIP
**K10**	Blood	15	CTX, TIC, TIM, PIP, TZP, CAZ, FEP, ATM, IPM, MEM, AMK, GEN, TOB, CIP, SXT
**K11**	Blood	13.5	CTX, TIC, TIM, PIP, TZP, CAZ, FEP, ATM, IPM, MEM, AMK, GEN, TOB, **CIP**
**K12**	Urine	15	CTX, TIC, TIM, PIP, TZP, CAZ, FEP, ATM, IPM, MEM, AMK, GEN, TOB, CIP, SXT
**K13**	Aspirate	7	CTX, TIC, PIP, CAZ, FEP, ATM, SXT
**K14**	Blood	15	CTX, TIC, TIM, PIP, TZP, CAZ, FEP, ATM, IPM, MEM, AMK, GEN, TOB, CIP, SXT
**K15**	Blood	7.5	CTX, TIC, **TIM**, PIP, CAZ, FEP, ATM, GEN
**K16**	Swab	7	CTX, TIC, **TIM**, PIP, CAZ, **FEP**, ATM, SXT
**K17**	Blood	14	CTX, TIC, TIM, PIP, TZP, CAZ, FEP, ATM, IPM, MEM, AMK, GEN, TOB, SXT
**K18**	Blood	13.5	CTX, TIC, TIM, PIP, TZP, CAZ, FEP, ATM, IPM, MEM, **AMK**, TOB, CIP, SXT
**K19**	Swab	15	CTX, TIC, TIM, PIP, TZP, CAZ, FEP, ATM, IPM, MEM, AMK, GEN, TOB, CIP, SXT
**K20**	Aspirate	14.5	CTX, TIC, TIM, PIP, TZP, CAZ, FEP, ATM, IPM, MEM, AMK, **GEN**, TOB, CIP, SXT
**K21**	Blood	14	CTX, TIC, TIM, PIP, TZP, CAZ, FEP, ATM, IPM, MEM, AMK, GEN, TOB, CIP
**K22**	Blood	15	CTX, TIC, TIM, PIP, TZP, CAZ, FEP, ATM, IPM, MEM, AMK, GEN, TOB, CIP, SXT
**K23**	Swab	13.5	CTX, TIC, TIM, PIP, TZP, CAZ, FEP, ATM, **IPM**, AMK, GEN, TOB, CIP, SXT

^a^ BAL, bronchoalveolar lavage; ETT, endotracheal tube. ^b^ For the calculation of resistance score*,* any resistant call was given a score of 1, while intermediate resistance was given a score of 0.5. ^c^ CTX, cefotaxime; TIC, ticarcillin; TIM, ticarcillin/clavulanate; PIP, piperacillin; TZP, piperacillin/tazobactam; CAZ, ceftazidime; FEP, cefepime; ATM, aztreonam; IPM, imipenem; MEM, meropenem; AMK, amikacin; GEN, gentamicin; TOB, tobramycin; CIP, ciprofloxacin; SXT, sulfamethoxazole/trimethoprim. Antibiotics to which the tested isolates showed intermediate resistance are in bold format.

**Table 2:** Antimicrobial resistance profile of *bla*_CTX-M-IV_-harboring *K. pneumoniae* clinical isolates and their transformants.

*K. pneumoniae* Isolates	Cefotaxime MIC (μg/mL)	Fold Increase in Cefotaxime MIC ^a^	Resistance Profile ^b^
**K7**	1024	-	AMC, CAZ, IPM, CTX, FEP, DO, GEN, CIP
**Transformant of K7**	8	64	AMC, CAZ, CTX
**K14**	1024	-	AMC, CAZ, IPM, CTX, SXT, FEP, GEN, CIP
**Transformant of K14**	16	128	AMC, CAZ, CTX
**K23**	1024	-	AMC, CAZ, **IPM**, CTX, SXT, FEP, DO, GEN, CIP
**Transformant of K23**	4	32	AMC, CAZ, CTX

^a^ The recipient, *E. coli* DH5α, was cefotaxime sensitive with an MIC of 0.125 μg/mL. ^b^ AMC, amoxicillin/clavulanate; CAZ, ceftazidime; IPM, imipenem; CTX, cefotaxime; SXT, sulfamethoxazole/trimethoprim; FEP, cefepime; DO, doxycycline; CIP, ciprofloxacin; GEN, gentamicin. Antibiotics to which the tested isolates showed intermediate resistance are in bold format.

**Table 3:** Features, molecular typing, resistance profile, plasmid replicon types, and virulence-associated factors of ST383 *K. pneumoniae* K22 strain isolated from Egypt.

ST ^a^	CPS ^b^	LPS ^c^	*wzc-* allele ^d^	*wzi-* typing ^e^	Resistance Profile ^f^		Plasmid Replicon Type ^g^	Virulence ^h^	
					Antimicrobial Class	Antimicrobial Resistance Genes		Virulence Determinants	Heavy Metal Resistance
ST383	K51	O1	*wzc*-50	*wzi-*705	Phenicols	*catA1*, *catB3*	Col(KPHS6) ColRNAI Col440II IncFIB_K_ IncFIB IncHI1B IncFII_K_ IncL/M	*irp1* *fyuA* *ybtAEPQSTUX* *iutA* *iucABCD* *rmpA, rmpA2* *rmpC* *mrkABDFHIJ*	*terABCDEWXYZ* *arsABCDR* *pcoABCDRSE* *silABCEFGPRS*
					Sulphonamides	*sul1, sul2*			
					Fosfomycin	*fosA*			
					Trimethoprim	*dfrA5*			
					β-Lactams	*bla*_SHV-26_, *bla*_TEM-1_, *bla*_NDM-5_, *bla*_OXA-1_, *bla*_OXA-9_, *bla*_OXA-48_, *bla*_CTX-M-14b_, *bla*_CTX-M-15_			
					Aminoglycosides	*aph(6)-Id, aph(3″)-Ib, aph(3′)-VIb, aadA1, aph(3′)-VI, aac(6′)-Ib, armA*			
					Macrolides, lincosamides and streptogramin B	*mphE, msrE, mphA*			
					Fluoroquinolones	*aac(6′)-Ib-cr, qnrS1, oqxA, oqxB*			
					Tetracycline	*tetA*			

^a^ ST, sequence type, data obtained from MLST v 2.0; ^b^ CPS, capsular polysaccharide, K-typing determined by K-PAM in silico diagnostic tool; ^c^ LPS, lipopolysaccharides, O-typing determined by K-PAM in silico diagnostic tool; ^d^ typing of *wzc* as determined from Institut Pasteur database; ^e^ *wzi* typing as determined from Institut Pasteur database; ^f^ antimicrobial resistance genes as obtained from ResFinder v 4.1, CGE pipelines; ^g^ data represent plasmid incompatibility (Inc) group designations as determined by PlasmidFinder v 2.1, CGE pipelines; ^h^ data obtained from Institut Pasteur database.

## Data Availability

The whole genome shotgun sequence for *K. pneumoniae* strain K22 was deposited in the DDBJ/ENA/GenBank under Bioproject accession number PRJNA825651 (http://www.ncbi.nlm.nih.gov/bioproject/825651 (accessed on 12 April 2022)) and reference BioSample accession number SAMN27531886 (https://www.ncbi.nlm.nih.gov/biosample/27531886 (accessed on 12 April 2022)). The raw sequence data have been submitted to the Sequence Read Archive (SRA) under study accession number PRJNA825651 (https://www.ncbi.nlm.nih.gov/sra/PRJNA825651 (accessed on 12 April 2022)). The de novo assembly of the two plasmids pEGY22_CTX-M-14 and pEGY22_CTX-M-15 investigated in this study were deposited in the NCBI using Banklt tool under accession numbers ON261190 and ON261191, respectively.

## Supplementary Materials

The following supporting information can be downloaded at: https://www.mdpi.com/article/10.3390/microorganisms10061097/s1, Table S1: In vitro activity of the tested antimicrobial agents against *K. pneumoniae* clinical isolates, Table S2: Assembly statistics generated through WGS of *K. pneumoniae* strain K22 from Egypt, Figure S1: Agarose gel showing PCR amplification of *bla*_CTX-M-IV_ gene in five *K. pneumoniae* isolates. Lane M: DNA molecular weight marker (100-bp ladder). Lanes 1, 2, 3, 5, and 6 show the amplicon (501 bp) of *bla*_CTX-M-IV_ gene corresponding to K1, K7, K14, K22, and K23, respectively. Lane 4 exhibits a negative result corresponding to isolate K18.
