# Supplementary material for: Whole Genome Characterization of the High-Risk Clone ST383 Klebsiella pneumoniae with a Simultaneous Carriage of blaCTX-M-14 on IncL/M Plasmid and blaCTX-M-15 on Convergent IncHI1B/IncFIB Plasmid from Egypt
Source: Microorganisms. 2022 May 26;10(6):1097. doi: 10.3390/microorganisms10061097 (PMC9228323; doi:10.3390/microorganisms10061097)
Supplement: Supplementary file 1 [file microorganisms-10-01097-s001.zip › microorganisms-1742567-supplementary.pdf]

Supplementary Table S1: *In vitro* activity of the tested antimicrobial agents against *K. pneumoniae* clinical isolates.

Antimicrobial agent	MIC (µg/ml)			% Sensitive	% Intermediate	% Resistant
	MIC_{50}*	MIC_{90}*	MIC range			
**Cefotaxime**	1024	>1024	128->1024	0	0	100
**Ticarcillin**	≥128	≥128	≥128	0	0	100
**Ticarcillin/clavulanate**	≥128	≥128	16-≥128	8.7	8.7	82.6
**Piperacillin**	≥128	≥128	≥128	0	0	100
**Piperacillin/tazobactam**	≥128	≥128	≤4-≥128	17.4	0	82.6
**Ceftazidime**	≥64	≥64	4-≥64	0	0	100
**Cefepime**	≥64	≥64	2-≥64	0	4.3	95.7
**Aztreonam**	≥64	≥64	≤1-≥64	4.3	0	95.7
**Imipenem**	≥16	≥16	≤0.25-≥16	17.4	4.3	78.3
**Meropenem**	≥16	≥16	≤0.25-≥16	21.7	0	78.3
**Amikacin**	≥64	≥64	≤2-≥64	17.4	8.7	73.9
**Gentamicin**	≥16	≥16	≤1-≥16	13	4.4	82.6
**Tobramycin**	≥16	≥16	≤1 ≥16	13	0	87
**Ciprofloxacin**	≥4	≥4	≤0.25-≥4	26.1	8.7	65.2
**Sulfamethoxazole/trimethoprim**	≥256	≥256	≤16-≥256	26.1	0	73.9

*MIC_{50} and MIC_{90}: MICs (µg/ml) for 50% and 90% of the tested isolates, respectively.

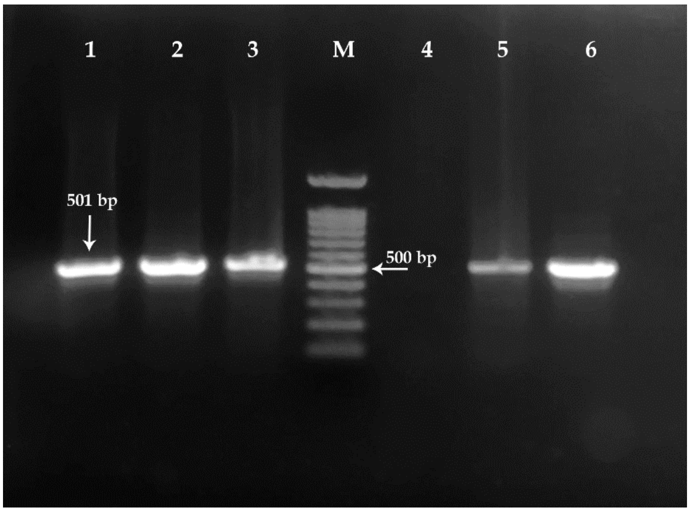

Supplementary Figure S1: Agarose gel showing PCR amplification of blaCTX-M-IV gene in five K. pneumoniae isolates. Lane M: DNA molecular weight marker (100-bp ladder). Lanes 1, 2, 3, 5, and 6 show the amplicon (501 bp) of blaCTX-M-IV gene corresponding to K1, K7, K14, K22, and K23, respectively. Lane 4 exhibits a negative result corresponding to isolate K18.

Supplementary Table S2: Assembly statistics generated through WGS of *K. pneumoniae* strain K22 from Egypt.

Depth of coverage	307X
Genome breadth (%)	99.1
Number of reads	5,420,196
Total length of sequences	6,329,505
Total number of contigs	295
N50 (bp)	88735
GC (%)	57.7
CDSs	2,915
5s, 16s, 23rrna	1, 1, 1
